# Decreased Polyunsaturated Fatty Acid Content Contributes to Increased Survival in Human Colon Cancer

**DOI:** 10.1155/2009/867915

**Published:** 2009-10-15

**Authors:** Manuela Oraldi, Antonella Trombetta, Fiorella Biasi, Rosa A. Canuto, Marina Maggiora, Giuliana Muzio

**Affiliations:** Dipartimento di Medicina ed Oncologia Sperimentale, Università di Torino, 10125 Torino, Italy

## Abstract

Among diet components, some fatty acids are known to affect several stages of colon carcinogenesis, whereas others are probably helpful in preventing tumors. In light of this, our aim was to determine the composition of fatty acids and the possible correlation with apoptosis in human colon carcinoma specimens at different Duke's stages and to evaluate the effect of enriching human colon cancer cell line with the possible reduced fatty acid(s). Specimens of carcinoma were compared with the corresponding non-neoplastic mucosa: a significant decrease of arachidonic acid, PPAR*α*, Bad, and Bax and a significant increase of COX-2, Bcl-2, and pBad were found. The importance of arachidonic acid in apoptosis was demonstrated by enriching a Caco-2 cell line with this fatty acid. It induced apoptosis in a dose- and time-dependent manner via induction of PPAR*α* that, in turn, decreased COX-2. In conclusion, the reduced content of arachidonic acid is likely related to carcinogenic process decreasing the susceptibility of cancer cells to apoptosis.

## 1. Introduction

Colon carcinoma is one of the world's most widespread tumors and appears to be correlated with diet [1]. Although the disease may evolve from genetic alterations in protooncogenes or tumor suppressor genes [2], there is evidence indicating that some dietary factors, such as fat and fiber, can also affect several stages of colon carcinogenesis [3, 4]. Among diet components, lipids are perceived as dangerous, although some lipids, such as certain polyunsaturated fatty acids (PUFAs), are considered to be positive for human health, preventing tumors and cardiovascular diseases [5–7]. Thus dietary adjustment aimed at preventing colorectal carcinoma is increasingly becoming an interesting option [8].

However, little is known about the composition of fatty acids in human colon carcinoma tissue versus that of non-neoplastic mucosa or about any changes in this composition during tumor development. Some findings have shown a decrease in PUFAs, such as linoleic and *α*-linolenic acids, together with an increase in free arachidonic acid, in colon cancer tissue versus those of a normal human large intestine, whereas other data have indicated an increase in linoleic acid and a decrease in *α*-linolenic and arachidonic acids [9–11].

More is known about the effect of PUFAs on cell lines and on the development of carcinoma in animals models. Supplementing cell lines with n-3 or n-6 PUFAs leads to an increased incorporation of these PUFAs in membrane phospholipids, influencing not only cell structure but also cell functions, including immunological response, proliferation, and survival [12–15]. Feeding animals with n-3 PUFAs has been found to inhibit tumor growth, probably because of the altered fatty acid composition [16, 17].

The effects of PUFAs on cell function and viability may be related to the fact that they and their metabolites are important mediators of the intracellular signal networks; that is, they may have an effect on gene expression after binding with peroxisome proliferator activated receptors (PPARs). PPARs are present in cells in three isoforms (*α*, *β*, *γ*) and play an important regulatory role in lipid metabolism and cancer development [15, 18, 19].

Among PUFA metabolites, the prostaglandines (PGs), which are derived from arachidonic acid, have been studied in depth. The PGE_2_ level has been shown to be significantly elevated during inflammation and in cancer, and this finding has been attributed to the increased expression of the inducible cyclo-oxygenase 2 (COX-2) [20]. The functions of COX-2-derived PGE_2_ in malignant and metastatic disease have been widely studied, and it appears to play a role in tumor invasion, in stimulating angiogenesis, and in modulating apoptosis [21, 22].

There is considerable controversy over the frequency of apoptotic cell death in the adenoma-carcinoma sequence in the human colon. Most researchers have found that the apoptotic index was higher in carcinoma than in normal colonic mucosa and adenomas, even though Tsujitani et al. reported a lower apoptotic rate in carcinoma, in a study comparing carcinomatous with adenomatous components in the same tumors [23, 24].

In light of the limited knowledge of the apoptotic events in colon cancer, and since PUFAs can modulate cancer development by regulating cell proliferation and/or viability, this study examined specimens of human colon carcinoma at different stages of Duke's classification and the corresponding non-neoplastic mucosa. The aim was to evaluate any changes in fatty acid content of phospholipids, in expression of some proteins regulating apoptosis and in PPAR*α*, whose fatty acids are ligands. COX-2, the enzyme responsible for the production of fatty acid metabolites active in cancer induction, was also analyzed.

To corroborate the results obtained in vivo, human colon carcinoma Caco-2 cells were used to evaluate the effect of arachidonic acid, an *ω*-6 fatty acid, in modulating apoptosis.

## 2. Materials and Methods

### 2.1. Colon Tissue Specimens

Colon adenocarcinoma and adjacent non-neoplastic colonic mucosa specimens were obtained from 28 surgical patients at the Oncology Center, Molinette Hospital, Turin, Italy. Adjacent connective tissue was stripped away in all cases, to ensure that there was no fat contamination, and colonic cancer specimens were free of non-neoplastic colon mucosa. Specimens were immediately frozen and stored at −80°C. Resected cancers were staged, according to Duke's classification, as A in 3 cases, B in 8 cases, C in 11 cases and D in 6 cases. 

Human samples were collected and analyzed with the full understanding and consent of the patient. Responsible Ethical Committee has approved these experiments.

### 2.2. Cell Cultures

Human colon cancer cells Caco-2 (ATCC, USA) were seeded (30 000 cells/cm^2^) and maintained for 48 hours in DMEM+GlutaMAX-1 medium containing 1% antibiotic/antimycotic solution (medium A), supplemented with 10% fetal bovine serum (FBS), and maintained at 37°C in a humidified atmosphere of 95% air and 5% CO2.

### 2.3. Caco-2 Cell Differentiation

Cells were maintained in culture for 2 and 20 days. After harvesting the cells were examined for arachidonic acid percentage content and for alkaline phosphatase (ALP) activity as differentiation marker. ALP activity was determined as in [25].

### 2.4. Arachidonic Acid Supplementation

A stock solution of free arachidonic acid (100 mM in FBS) was prepared and stored at −20°C until use. Twenty four hours after seeding undifferentiated cells, culture medium was removed and replaced by medium B (medium A plus 0.4% serum bovine albumin (fatty acid free), 1% vitamin solution, 1% nonessential amino acids) supplemented or not with arachidonic acid (time 0). Arachidonic acid stock solution was diluted to the final concentration directly in medium B, used to replace medium A. FBS alone was added to control cells.

At the experimental times indicated in the figures, Caco-2 cells, treated or not with arachidonic acid, were trypsinized after collecting culture medium, harvested and centrifuged at 600 g for 10 minutes, to determine apoptosis parameters.

### 2.5. Treatment of Cells with PPAR*α* Antagonist MK886 or with COX-2 Inhibitor NS398

Caco-2 cells were treated with arachidonic acid, as described above, and with the PPAR*α* antagonist MK886 or COX-2 inhibitor NS398. 2 *μ*M MK886 was administered to the cells when arachidonic acid was added (time 0), and 50 *μ*M NS398 one hour before arachidonic acid addition.

### 2.6. Apoptosis Evaluation

Percentages of viable, necrotic, or apoptotic cells were evaluated by determining the DNA content after propidium iodide staining in flowcytometry as described in [18].

The presence of apoptotic cells was also shown by staining cells with DAPI (4′, 6-diamidino 2-phenylindole) then observing them using fluorescence microscopy.

### 2.7. Analysis of Fatty Acid Composition in Phospholipids

The percentage content of different fatty acids was determined in the polar lipids extracted from non-neoplastic and colon cancer specimens and from Caco-2 cells as described in [19]. The percentage of single fatty acids was calculated taking the content of total fatty acids in phospholipids as 100%. Methyl heptadecanoate was added to each preparation as internal standard.

### 2.8. Western Blot Analysis

For each colon specimen, about 50 mg of tissue was washed twice in cold PBS and then homogenized by sonication in 150 *μ*L of Totex buffer (pH 7.9) containing 20 mM Hepes, 350 mM NaCl, 20% glycerol, 1% NP-40 substitute, 1 mM MgCl_2_, 0.5 mM EDTA, 0.1 mM EGTA, 1 mM Na-orthovanadate, 1 mM phenyl methyl sulfonyl fluoride (PMSF), 5 mM DL-Dithiothreitol, and 15 *μ*g/mL leupeptin. The homogenates were kept on ice for 1 hour and then centrifuged in a microfuge at 13500 rpm for 25 minutes. Supernatants containing the extracted proteins were then collected and used for Western blot analysis as described in [18].

The membranes were incubated with polyclonal anti-PPAR*α*, Bcl-2, pBAD, BAD and Bak, with monoclonal anti-COX-2 (all purchased from Santa Cruz Biotechnology, Heidelberg, Germany), or anti-*β*-actin (purchased from Sigma, Mo, USA) antibodies. The densitometry value for each protein was referred to the corresponding *β*-actin value.

Collected Caco-2 cells were suspended to 50% (w/v) in lysis buffer containing 20 mM Tris-HCl (pH 7.4), 150 mM NaCl, 5 mM EDTA, 1% NP-40 substitute, 1 mM PMSF, 1 mM Na-orthovanadate, and 15 *μ*g/mL leupeptin. Cells were then incubated for 30 minutes on ice and sonicated 3 times for 3 seconds. After centrifugation, the extracted proteins were used for Western blot analysis as described above. PPAR*α*, Bcl-2, Bak, and COX-2 were evaluated.

### 2.9. Protein Determination

Protein content was determined with the Protein Assay Kit 2 (BIO-RAD Laboratories, CA, USA).

### 2.10. Statistical Analysis

Data for fatty acid percentage content in cancer and corresponding mucosa specimens are expressed as means ± SD. Significant differences between all cancer and their corresponding mucosa specimens, and those among cancer groups at different stages of Duke's classification, were assessed by variance analysis, followed by the *post hoc* Newman-Keuls test.

Protein content data are expressed as means ± SD, calculated by relating the values in cancer specimens to those in mucosa ones, taken as 1. Significant differences between cancer and the corresponding mucosa specimens were assessed by the Wilcoxon signed rank test; significant differences among cancer groups at different stages of Duke's classification were assessed by variance analysis, followed by the *post-hoc* Newman-Keuls test.

Significant differences between Caco-2 cells treated with arachidonic acid or untreated were assessed by variance analysis, followed by *post hoc* Newman-Keuls test.

## 3. Results

### 3.1. Human Colon Cancer Specimens

#### 3.1.1. Colon Cancer Classification

Colon non-neoplastic mucosa and carcinoma specimens were obtained from 28 surgical patients (15 women, 13 men; age range 32 to 87 years; mean 67.50 ± 11.69). Carcinomas ranged from well to poorly differentiated and were at the following stages of Duke's classification: 3 cases of resected cancers were in stage A, 8 cases in B, 11 cases in C, and 6 cases in D.

#### 3.1.2. Fatty Acid Composition in Phospholipids

Table 1 reports the percentage of the content of various PUFAs in phospholipids extracted from cancer specimens at different Duke's stages and from the corresponding non-neoplastic mucosa. Table 1 also reports the sum (∑) of PUFAs and of monounsaturated fatty acids (MUFAs) plus saturated fatty acids. The table also shows the significance of differences between mucosa and tumor specimens; the significance among tumor specimens at different Duke's stages was not reported, since there were no differences.

Comparing the percentage of the content of each PUFA in cancer specimens and in the corresponding mucosa, a significant decrease was seen in arachidonic acid at all Duke's stages, for linoleic acid at stage D, and for CLA at stages B, C, and D. On the contrary, no difference was found for linolenic or docosaexaenoic acids. The decreases in linoleic and arachidonic acids and in CLA accounted for the decreased ∑ PUFA content observed at all Duke's stages.

#### 3.1.3. COX-2 Content

**Figure 1:**
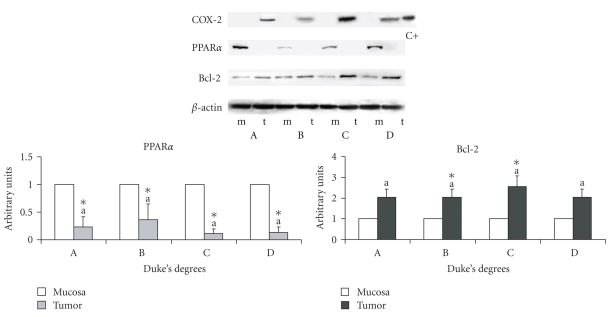
Protein content of COX-2, PPAR*α*, and Bcl-2 in cancer specimens and corresponding non-neoplastic mucosa. The densitometry value for PPAR*α* and Bcl-2, referred to the corresponding *β*-actin value, was compared with that of the corresponding non-neoplastic mucosa, taken as 1. The data are expressed in means ± SD. Since COX-2 was not expressed in all mucosa specimens, the same comparative data as for PPAR*α* is not available. Significant differences between cancer specimens and relative mucosa specimens were assessed by the Wilcoxon signed rank test (*P < .05). Significant differences among cancer groups at different Duke's stages were assessed by variance analysis followed by the *post hoc* Newman-Keuls test. C+: positive control.

In view of the decreased arachidonic acid percentage, the protein content of COX-2 was evaluated. It was found to be increased in cancer specimens versus mucosa specimens. Figure 1 shows a typical COX-2 Western blot: the protein was not expressed in non-neoplastic mucosa and was increased in carcinoma at all stages. Since COX-2 was not expressed in all mucosa specimens, the same comparative data are not available for this protein.

#### 3.1.4. PPAR*α* Content

Since fatty acids and their metabolites are preferential ligands of PPAR*α*, the protein content of this nuclear receptor was evaluated (Figure 1), showing a decrease in carcinoma versus mucosa at all Duke's stages with no significant differences among Duke's stages.

#### 3.1.5. Apoptosis Parameters

**Figure 2:**
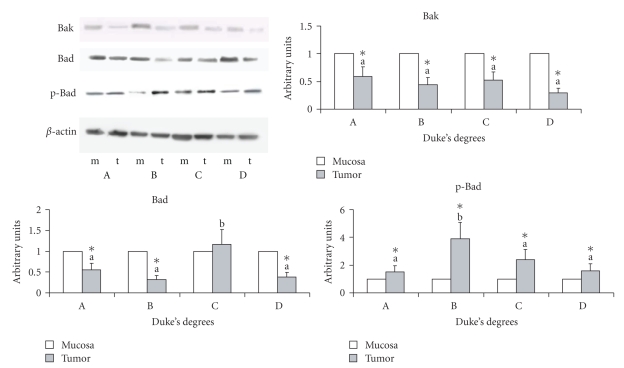
Protein content of Bak, Bad, and p-Bad in cancer specimens and corresponding non-neoplastic mucosa specimens. The densitometry values, the data, and significant differences are as in Figure 1.

The antiapoptotic protein Bcl-2 and inactive phosphorylated form of Bad (Figures 1 and 2) were increased in all cancer specimens, with no differences among Duke's stages, whereas the proapototic proteins Bad and Bak were decreased (Figure 2).

### 3.2. Human Colon Cancer Cells Caco-2

#### 3.2.1. Arachidonic Acid Content

**Figure 3:**
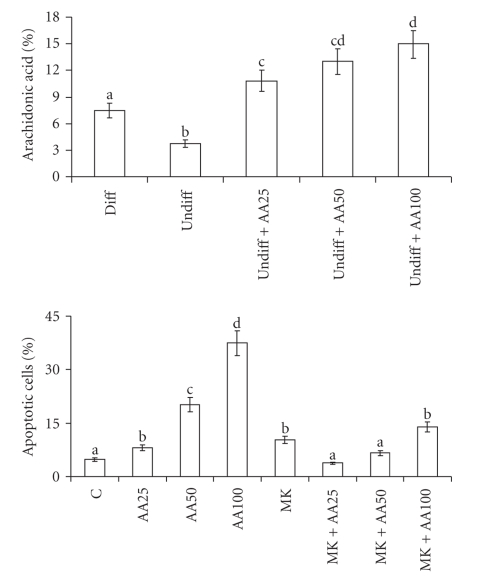
Percentage of the content of arachidonic acid and percentage of cells in apoptosis in human Caco-2 cells 48 hours after enrichment with arachidonic acid. Data are expressed in means ± SD of 4 experiments. For arachidonic acid and for apoptosis means with different letters are significantly different from one another (*P* < .05), as determined by variance analysis followed by *post hoc* Newman-Keuls test. For percentage of the content of arachidonic acid; diff: differentiated Caco-2 cells; undiff: undifferentiated Caco-2 cells; undiff + AA25: undifferentiated Caco-2 cells treated with 25 *μ*M arachidonic acid; undiff + AA50: undifferentiated Caco-2 cells treated with 50 *μ*M arachidonic acid; undiff + AA100: undifferentiated Caco-2 cells treated with 100 *μ*M arachidonic acid. For percentage of cells in apoptosis: C: control cells; AA25: cells treated with 25 *μ*M arachidonic acid; AA50: cells treated with 50 *μ*M arachidonic acid; AA100: cells treated with 100 *μ*M arachidonic acid; MK: cells treated with 2 *μ*M MK886.

Figure 3 shows the percentage content of fatty acids in phospholipids extracted from differentiated and undifferentiated Caco-2 cells, showing that arachidonic acid is present in higher percentage content in differentiated cells than in undifferentiated cells (3.7 versus 9.4, resp.). Enriching undifferentiated cells with 25, 50, or 100 *μ*M arachidonic acid, the percentage content of this fatty acid increased reaching a value of 15 with 100 *μ*M arachidonic acid.

#### 3.2.2. Apoptosis and PPAR*α* Content

**Figure 4:**
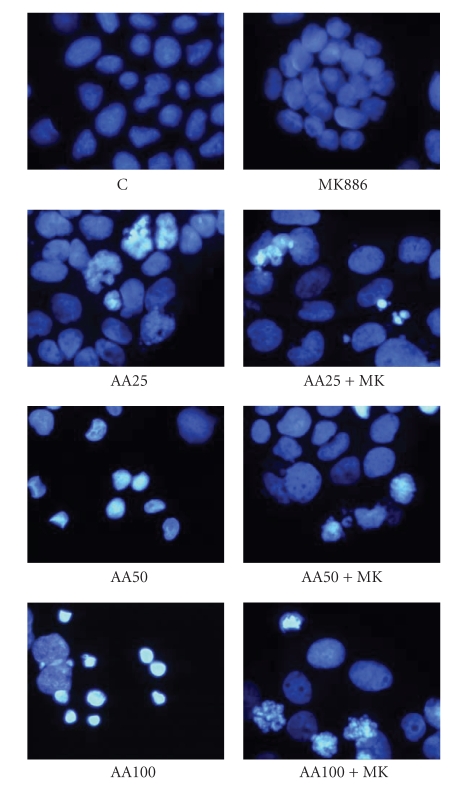
Apoptosis in human Caco-2 cells 48 hours after enrichment with arachidonic acid. Apoptosis was determined by staining the cells with DAPI. C: control cells; AA25: cells treated with 25 *μ*M arachidonic acid; AA50: cells treated with 50 *μ*M arachidonic acid; AA100: cells treated with 100 *μ*M arachidonic acid; MK: cells treated with 2 *μ*M MK886.

The increased arachidonic acid content in treated-cells affected cell viability, inducing apoptosis, as shown in Figure 3. Apoptosis was also shown by DAPI staining (Figure 4): arachidonic acid-treated cells showed fragmented and condensed nuclei, and apoptotic bodies; on the contrary, in untreated cells few apoptotic figures were observed.

**Figure 5:**
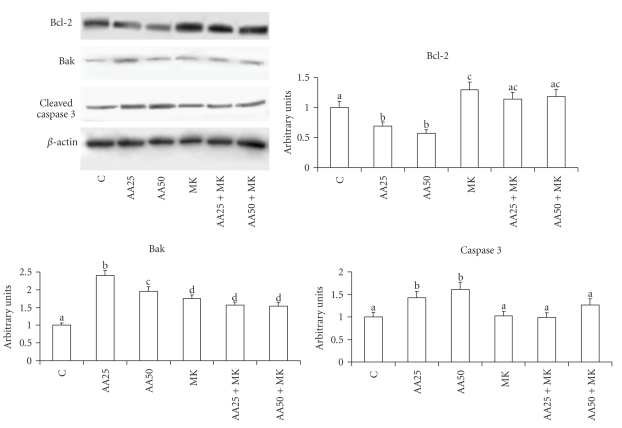
Protein content of Bcl-2, Bak, and caspase 3 in Caco-2 cells 48 hours after enrichment with arachidonic acid. Data are expressed in means ± SD of 4 experiments. Means with different letters are significantly different from one another (*P* < .05), as determined by variance analysis followed by *post hoc* Newman-Keuls test. The densitometry values, referred to as the corresponding *β*-actin value, were compared with those of control cells, taken as 1. C: control cells; AA25: cells treated with 25 *μ*M arachidonic acid; AA50: cells treated with 50 *μ*M arachidonic acid; MK: cells treated with 2 *μ*M MK886.

The involvement of arachidonic acid in modulating apoptosis was confirmed by analyzing the antiapoptotic protein Bcl-2 and the proapoptotic proteins Bak and caspase 3. Figure 5 shows a decrease of the first protein and an increase of the second ones in arachidonic-treated cells in comparison with control cells. Caspase 3 induction was shown by an increased amount of cleaved form.

**Figure 6:**
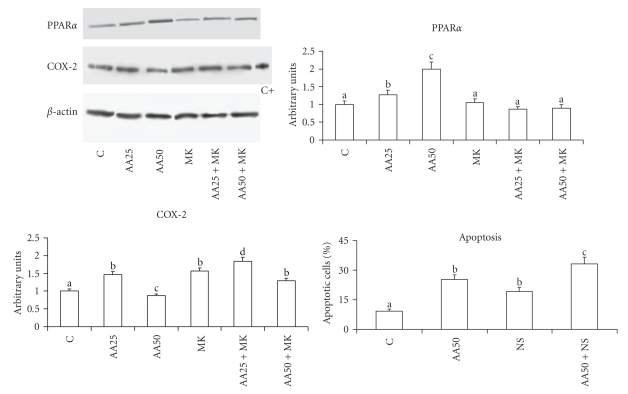
Protein content of PPAR*α* and COX-2 and percentage of cells in apoptosis in human Caco-2 cells 48 hours after enrichment with arachidonic acid and COX-2 inhibitor NS398. Data are expressed in means ± SD of 3 experiments. For COX-2 and for apoptosis means with different letters are significantly different from one another (*P* < .05), as determined by variance analysis followed by *post hoc* Newman-Keuls test. For PPAR*α* and COX-2, the densitometry values, referred to as the corresponding *β*-actin value, was compared with those of control cells, taken as 1. C: control cells; AA25: cells treated with 25 *μ*M arachidonic acid; AA50: cells treated with 50 *μ*M arachidonic acid; MK: cells treated with 2 *μ*M MK886. For percentage of cells in apoptosis, C: control cells; AA50: cells treated with 50 *μ*M arachidonic acid; NS: cells treated with 50 *μ*M NS398; AA50 + NS: cells treated with 50 *μ*M arachidonic acid plus 50 *μ*M NS398.

Figure 6 reports the PPAR*α* content, demonstrating an increase of this nuclear receptor in treated cells. Its involvement in arachidonic acid-induced apoptosis was demonstrated by treating the cells with the specific PPAR*α* antagonist MK886. Figures 3 and 4 show that MK886 prevented apoptosis induction and growth inhibition due to arachidonic acid. The reverting effect of MK886 was also confirmed by DAPI staining. The prevention of apoptosis induction was further confirmed by evaluation of anti- and proapoptotic proteins (Figure 5). Bcl-2 level was increased in the cells treated with arachidonic acid and MK886 in comparison with the cells treated with arachidonic acid alone, whereas Bak was decreased.

#### 3.2.3. COX-2 Content

The evaluation of COX-2 in Caco-2 cells enriched with arachidonic acid showed that the lowest concentration of fatty acid slowly increased the enzyme content, whereas the highest concentration decreased it (Figure 6). PPAR*α* antagonist MK886, which did not induce apoptosis, increased COX-2 content. To demonstrate that COX-2 was involved in blocking apoptosis, specific COX-2 inhibitor NS398 was used, showing that it increased apoptotic cell percentage in comparison with untreated cells. Cells treated with both arachidonic acid and COX-2 inhibitor showed a further increase in apoptosis (Figure 6).

## 4. Discussion

The study is focused on the fatty acid content in phospholipids of human colon cancer samples of different degrees of malignancy and on some factors regulating apoptosis. The observations suggested by the results obtained with cancer specimens were confirmed using “in vitro” experiments using Caco-2 cells.

The most significant differences between colon cancer specimens and the corresponding non-neoplastic mucosa were as follows: (1) the percentage of the content of ∑ PUFAs, linoleic acid, CLA, and arachidonic acid decreased; (2) COX-2 increased in cancer specimens, whereas in non-neoplastic mucosa it was not expressed; (3) PPAR*α* decreased; (4) the antiapoptotic proteins Bcl-2 and pBad increased, whereas the proapototic proteins Bad and Bak decreased.

The results in cancer specimens were also correlated with the patients' clinical stage but showed no evidence that the clinical stage influenced the parameters examined. In view of this, it is possible to hypothesize that changes in some parameters, such as PUFA and proteins involved in apoptosis, are important for the entire carcinogenesis process.

The low arachidonic acid content present in colon cancer was also evident in undifferentiated Caco-2 cells. It is noteworthy that the content of this fatty acid in Caco-2 cells is in relation to the degree of deviation. In fact, its percentage was lower in undifferentiated cells than in differentiated cells, pointing again to the importance of arachidonic acid in the development of tumours.

The reduced content of arachidonic acid might be also considered a defense mechanism whereby cancer cells protect themselves from the cytotoxic effect of this compound. In fact, the low level of arachidonic acid was accompanied by an increase in antiapoptotic protein Bcl-2 and by the reduction of proapototic proteins and PPAR*α*, indicating a decreased susceptibility to apoptosis in colon cancer with respect to non-neoplastic mucosa. This correlation between arachidonic acid and susceptibility to apoptosis, to which these “in vivo” results point, was supported by “in vitro” experiments, in which exogenously added arachidonic acid was found to cause apoptosis in colon cancer Caco-2 cells. Cell death was found to be correlated with the intracellular level of arachidonic acid. The induction of apoptosis by arachidonic acid has also been confirmed by other authors [26].

The decreased percentage of arachidonic acid in tumors at all Duke's stages was probably correlated with the increased expression of COX-2. Previous studies have shown this enzyme to be increased in colon and rectal cancer [27], and to not be expressed in non-neoplastic mucosa [27]. The importance of COX-2 in modulating colon carcinogenesis has been shown by the observation that NSAIDs prevent colon cancer and cause apoptosis [28]. The apoptosis-inducing mechanism might result from the accumulation of the COX substrate, arachidonic acid, which is a strong signal for induction of apoptosis: COX-2 may promote carcinogenesis by lowering the arachidonic acid level and producing PGE_2_ [29]. Also “in vitro” results shown here confirmed that COX-2 inhibitor induces apoptosis.

The mechanism triggered by arachidonic acid which leads to apoptosis may be ascribed to PPAR*α* modulation. In fact, the decreased PPAR*α* in tumor specimens compared to non-neoplastic mucosa was supported by our “in vitro” study supporting a direct correlation between PPAR*α* and apoptosis. The inverse correlation between PPAR*α* and COX-2 shown “in vivo” was confirmed by “in vitro” experiments, where the highest arachidonic acid concentration, determining the highest PPAR*α* content, was accompanied by a decrease of COX-2 content. This observation supports with other authors' studies reporting that PPAR*α* ligands inhibit COX-2 expression by directly antagonizing NFkB- and AP-1-mediated signalling pathways [30].

The decrease of PPAR*α* in tumors confirms the observation of other authors that a significant decrease in its expression is found in human tubular adenomas compared to normal human colonic epithelial cells [29]. Moreover, the importance of PPAR*α* in apoptosis has also been seen by other “in vitro” studies [18, 31], stimulating interest in the investigation of PPAR*α* as a possible therapeutic target to prevent colon cancer formation.

## 5. Conclusions

The bulk of both “in vivo” and “in vitro” results strongly suggests that colon cancer cells are characterized by decreased susceptibility to apoptosis deriving from decreased arachidonic acid content, PPAR*α* and proapoptotic proteins, and increased antiapoptotic proteins and COX-2.

In fact, colon cancer in humans shows a modified fatty acid composition in phospholipids, with a reduction in arachidonic acid content. These changes are associated with an increase in survival-related parameters. The reduced content of arachidonic acid is likely related to the carcinogenic process and protects against the effects of oxidative stress products, decreasing the susceptibility of cancer cells to apoptosis. This last observation was supported by treating Caco-2 cells with arachidonic acid.

## Figures and Tables

**Table 1:** Percentage of the content of polyunsaturated fatty acids in phospholipids extracted from colon cancer specimens and corresponding non-neoplastic mucosa.

Fatty acids	A		B		C		D	
	Mucosa	Tumour	Mucosa	Tumour	Mucosa	Tumour	Mucosa	Tumour
18 : 2	12.5	9.1	10.1	8.9	12.1	10.7	12.5	7.6*
18 : 3	0.2	0.7	0.4	0.3	1.0	1.1	0.8	0.6
CLA	0.5	0.5	1.4	0.2*	1.2	0.5*	0.7	0.2*
20 : 4	11.0	7.1*	11.4	8.1*	11.6	7.7*	13.4	9.5*
22 : 6	1.0	1.5	1.8	1.5	1.6	1.8	2.4	1.9
∑ PUFA	25.2	18.9*	25.1	19.0*	27.6	21.3*	29.9	19.8*
∑ MUFA + saturated	74.8	81.1*	74.9	81.1*	72.4	78.7*	70.1	80.2*

The SD was not more than 10%. For each fatty acid, the variance analysis followed by *post hoc* Newman-Keuls test was carried out. The table only shows the significance of differences between mucosa and tumor specimens (∗ tumour versus mucosa, *P* < .05). The differences among tumour specimens at different Duke's stages (A, B, C, D) were not significant. ∑ PUFA: sum of all polyunsaturated fatty acids; ∑ MUFA + saturated: sum of all monounsaturated and saturated fatty acids.
